# The Care of Children's Backs

**Published:** 1912-03-23

**Authors:** 


### MATERNITY AND MOTHERING.

(Inquiries and Correspondence are invited.)

### The Care of Children's Backs.

In everyday life it is well recognised that prevention is better than cure, though careless people often will not bother themselves to think out details and methods of prevention. In questions of health this old proverb applies with especial force; the preventive medicine of the day is having its full recognition in the State services, but it is not always realised that the individual and the family should practise it too. Parental care too often consists in fussiness about non-essentials, to the neglect of those really important matters upon which the future well-being of the child so much depends.

In particular certain deformities, which require months or years of patient treatment to cure, are largely a matter of inadequate supervision and are strictly preventable if instructed care be exercised. The back is an excellent case in point. Large numbers of children suffer from greater or smaller degrees of deformity of the back, and in by far the great majority these could have been avoided by proper precautions. This statement applies in some degree even to deformities due to definite bone disease, such as rickets or tuberculosis of the vertebræ; but it is not to this kind of deformity that attention is directed in what follows. The ordinary crooked back arises primarily from muscular weakness; and it is only after a long period of such weakness that any structural change takes place in the bones of a nature to cause permanent crookedness of the back.

When infantile paralysis, post-diphtheritic neuritis, or any similar acute illness causing serious and lasting paralysis or weakness has invalided a child, the resulting muscular deficiency is usually so great as to attract immediate attention and to receive careful treatment. It is rather in those cases where a slight debility from adenoids, mild rickets, or other minor ailment is the cause of muscular insufficiency that the development of spinal deformity is insidious and unnoticed.

Nor are the exciting causes of "crooked backs" confined to identifiable morbid conditions such as those mentioned. Sometimes none of these intrinsic factors can be traced, and indeed there are extrinsic influences almost more important still. Among these may be mentioned faulty posture, whether due to an ill-designed seat or desk in the school-room, to faults of dress or of occupation. Some authors hold that an infant's spine may become curved if the nurse or mother carries it always on the same arm; but this is a doubtful point. Sometimes a congenital abnormality, such as an extra rib, affects the shape of the back; sometimes a remote lesion, such as a shortened leg or thigh or a tilted pelvis, affects it secondarily. In any case the attention of parents or others in charge of children is generally only aroused when the mischief has already become considerable. A marked inequality in the height of the shoulders may be the first thing noticed, and even more commonly still a projecting shoulder-blade arouses attention; in the latter event it is usually said that the "shoulder is growing out."

Whenever matters have got to this pitch, inspection will usually reveal the presence of scoliosis or of kyphosis, or of both. These are the scientific names for a crooked spine; the former implies a bending to one or other side, the latter a bending forwards or stoop. Such inspection must be thorough and complete. The whole of the back should be entirely uncovered, right down to below the level of the hips. If the latter be actually tilted, there is bound to be some scoliosis; unless, of course, the patient is standing with one knee bent. Slight degrees of crookedness of the back require skill in detection, and may easily escape the notice of anyone but a competent doctor. Moreover, when the deformity has been detected, only a medical man can determine to what cause it is due.

Assuming however that it has been discovered that a child has some deformity of the back, not due to any bone disease such as tuberculosis, and not of such long standing as to involve changes in the shape of the bones, the line of treatment which will prove best for such a child is now fairly well established. Food must be plain but nourishing, sufficient in quantity but not allowed to excess, and taken at regular intervals. Mastication is to be ensured by dental treatment of any defective teeth. Proper hours of sleep are to be prescribed, and no reading in bed or other curtailment of those hours permitted. Abundance of fresh air, and of exercise in the fresh air, is to be insisted on: the studious child who would rather sit poring over a book than go out to play is especially to be encouraged to take outdoor exercise. Overwork, either in school or at home lessons, is to be avoided; and this applies to girls even more than to boys, particularly when pubescent. In short, the teachings of modern hygiene are to be implicitly followed.

Besides this it may be, very often will be, necessary to conduct a more direct attack upon the trouble. Broadly speaking, this consists of two parts: gymnastic exercises under supervision, and artificial supports. The former are often sufficient of themselves; the latter should never be ordered for weak backs of the sort now under discussion without at the same time resorting to gymnastics. Such exercises must, to be of any value, be performed under almost constant skilled supervision. The system was originally introduced from Sweden; but orthopædic surgeons have modified the original Swedish system in several respects, and the exercises enjoined to-day are a composite product in which the experience of numerous specialists has been incorporated. In America, the use of light plaster-of-Paris jackets is now being advocated for bad cases, frequently changed as the back gradually returns towards a normal shape. Patience and perseverance on the part of those in charge of the child are essential; but with them, the chances are strongly in favour of complete recovery in these cases.

